# ACE2 protein expression in lung tissues of severe COVID-19 infection

**DOI:** 10.1038/s41598-022-07918-6

**Published:** 2022-03-08

**Authors:** Atish Gheware, Animesh Ray, Deeksha Rana, Prashant Bajpai, Aruna Nambirajan, S. Arulselvi, Purva Mathur, Anjan Trikha, Sudheer Arava, Prasenjit Das, Asit Ranjan Mridha, Geetika Singh, Manish Soneja, Neeraj Nischal, Sanjeev Lalwani, Naveet Wig, Chitra Sarkar, Deepali Jain

**Affiliations:** 1 Department of Pathology, All India Institute of Medical Sciences, New Delhi, 110029 India; 2 Department of Medicine, All India Institute of Medical Sciences, New Delhi, 110029 India; 3 Emory Vaccine Center, International Center for Genetic Engineering and Biotechnology, New Delhi, 110067 India; 4 Department of Laboratory Medicine, JPNATC, All India Institute of Medical Sciences, New Delhi, 110029 India; 5 Department of Anaesthesiology, Critical Care and Pain Medicine, All India Institute of Medical Sciences, New Delhi, 110029 India; 6 Division of Forensic Pathology and Molecular Laboratory, All India Institute of Medical Sciences, New Delhi, 110029 India

**Keywords:** Molecular biology, Biomarkers, Diseases, Medical research, Pathogenesis, Risk factors

## Abstract

Angiotensin-converting enzyme 2 (ACE2) is a key host protein by which severe acute respiratory syndrome coronavirus-2 (SARS-CoV-2) enters and multiplies within cells. The level of ACE2 expression in the lung is hypothesised to correlate with an increased risk of severe infection and complications in COrona VIrus Disease 2019 (COVID-19). To test this hypothesis, we compared the protein expression status of ACE2 by immunohistochemistry (IHC) in post-mortem lung samples of patients who died of severe COVID-19 and lung samples obtained from non-COVID-19 patients for other indications. IHC for CD61 and CD163 was performed for the assessment of platelet-rich microthrombi and macrophages, respectively. IHC for SARS-CoV-2 viral antigen was also performed. In a total of 55, 44 COVID-19 post-mortem lung samples were tested for ACE2, 36 for CD163, and 26 for CD61, compared to 15 non-covid 19 control lung sections. Quantification of immunostaining, random sampling, and correlation analysis were used to substantiate the morphologic findings. Our results show that ACE2 protein expression was significantly higher in COVID-19 post-mortem lung tissues than in controls, regardless of sample size. Histomorphology in COVID-19 lungs showed diffuse alveolar damage (DAD), acute bronchopneumonia, and acute lung injury with SARS-CoV-2 viral protein detected in a subset of cases. ACE2 expression levels were positively correlated with increased expression levels of CD61 and CD163. In conclusion, our results show significantly higher ACE2 protein expression in severe COVID-19 disease, correlating with increased macrophage infiltration and microthrombi, suggesting a pathobiological role in disease severity.

## Introduction

The pneumonia epidemic COrona VIrus Disease 2019 (COVID-19) triggered by severe acute respiratory syndrome coronavirus- 2 (SARS-CoV-2) is spreading at an alarming rate^1,2^. The membrane-bound angiotensin-converting enzyme-2 (ACE-2), which is expressed in various human organs, has been identified as the SARS-CoV-2 entry receptor^3,4^. ACE2 is a negative regulator of the renin-angiotensin pathway. It inhibits the effects of angiotensin-II (Ang II) and its structurally related receptor ACE, thereby negatively regulating vasoconstriction, cell proliferation, and inflammation^5,6^. The role of ACE2 expression in COVID-19 disease pathogenesis is not well understood, and whether its level relates to the risk of infection or disease severity is not known. At the transcriptional level, ACE2 mRNA is normally detected at low levels in the respiratory system^6,7^. In COVID-19 disease, studies have shown higher ACE2 mRNA levels in patients’ nasal swabs, bronchial brushes, and bronchoalveolar lavages^8^ though more recent in-vitro studies did not find any such ACE2 mRNA elevation^9,10^. Immunohistochemistry (IHC) analysis of lungs from a small number of COVID-19 patients revealed a higher number of alveolar epithelial cells expressing ACE2 protein^11,12^. This is in contrast to a cell culture study that demonstrated decreased ACE2 protein levels after 24 h of infection in SARS-CoV-2 transfected cells^13^. Thus, while it appears that ACE2 mRNA levels rise or stay stable during SARS-CoV2 infection, protein levels remain a point of contention. As a result, the status of the ACE2 protein remains uncertain in the COVID-19 setting. Given the current scenario where individual risk factors for severe COVID-19 disease still remain unclear, this cross-sectional observational study aimed to determine the expression of ACE2 in the lung parenchyma of post-mortem tissue from fatal COVID-19 cases in relation to histopathology, markers of vascular and inflammatory responses, and clinical features in order to ascertain its role, if any, in disease severity and potential for therapeutic targeting.

## Results

### Patients’ clinical data and pathological finding

A total of 55 lung samples obtained from deceased COVID-19 patients were included. Patients included 38 men and 17 women, with a median age of 49 years (SD 17.27; range 13–82) at death. All patients tested positive for SARS-CoV-2 using nasopharyngeal swab PCR. Shortness of breath (49%), fever (40%), and cough (21.8%) were the most common symptoms at onset. Status regarding time spent in the hospital was available for 36 patients, and the mean length of stay of these patients in the critical care unit or intermediate medical ward was 12.6 days. Respiratory failure or multiorgan failure involving the respiratory system was the leading cause of death. In terms of previous comorbidities, 11 (20%) had diabetes, 15 (27.27%) had cardiovascular complications (including hypertension in 14 patients), 9 (16.3%) had previous respiratory illnesses, 18 (32.7%) had liver or renal diseases, and five (9%) had cancer. Additionally, 22 patients had other complications such as hypothyroidism, anaemia, steroid-induced hypoglycaemia, or dyslipidaemia (Table 1, supplemental Fig. S1).

**Table 1:** Patient clinicopathological information.

Characteristics	*N* or mean	% or range
**COVID-19 cohort (55 patients)**		
Age	46 y	13–82 y
Sex	M 38, F17 (2.2:1)	
Hospitalisation time	12.6 d	1–88 d
**Major symptoms on admission**		
Fever	22	40.00
Cough	12	21.82
Shortness of breath	27	49.09
**Major comorbidities**		
Diabetes	11	20.00
Cardiovascular complications	15	27.27
Respiratory complications	9	16.36
Cancer	5	9.09
Liver or kidney complications	18	32.73
Thyroid complications	5	9.09
Aplastic anaemia	2	3.64
Patients with no prior comorbidities	4	7.27
**Most common lung pathological features**		
Exudative phase of diffuse alveolar damage	25	45.45
Organizing phase of diffuse alveolar damage	13	23.64
Acute bronchopneumonia	5	9.09
Acute lung injury	9	16.36
No changes	3	5.45
Coexisting acute bronchopneumonia with DAD	12	
**Control cohort (15 subjects)**		
Age	43.1 y	18–70 y
Sex	M 8, F 7 (1:1)	
**Lung pathology**		
Normal lung	13	86.66
Diffuse alveolar haemorrhage	1	6.66
Diffuse alveolar damage	1	6.66

**Figure 1:**
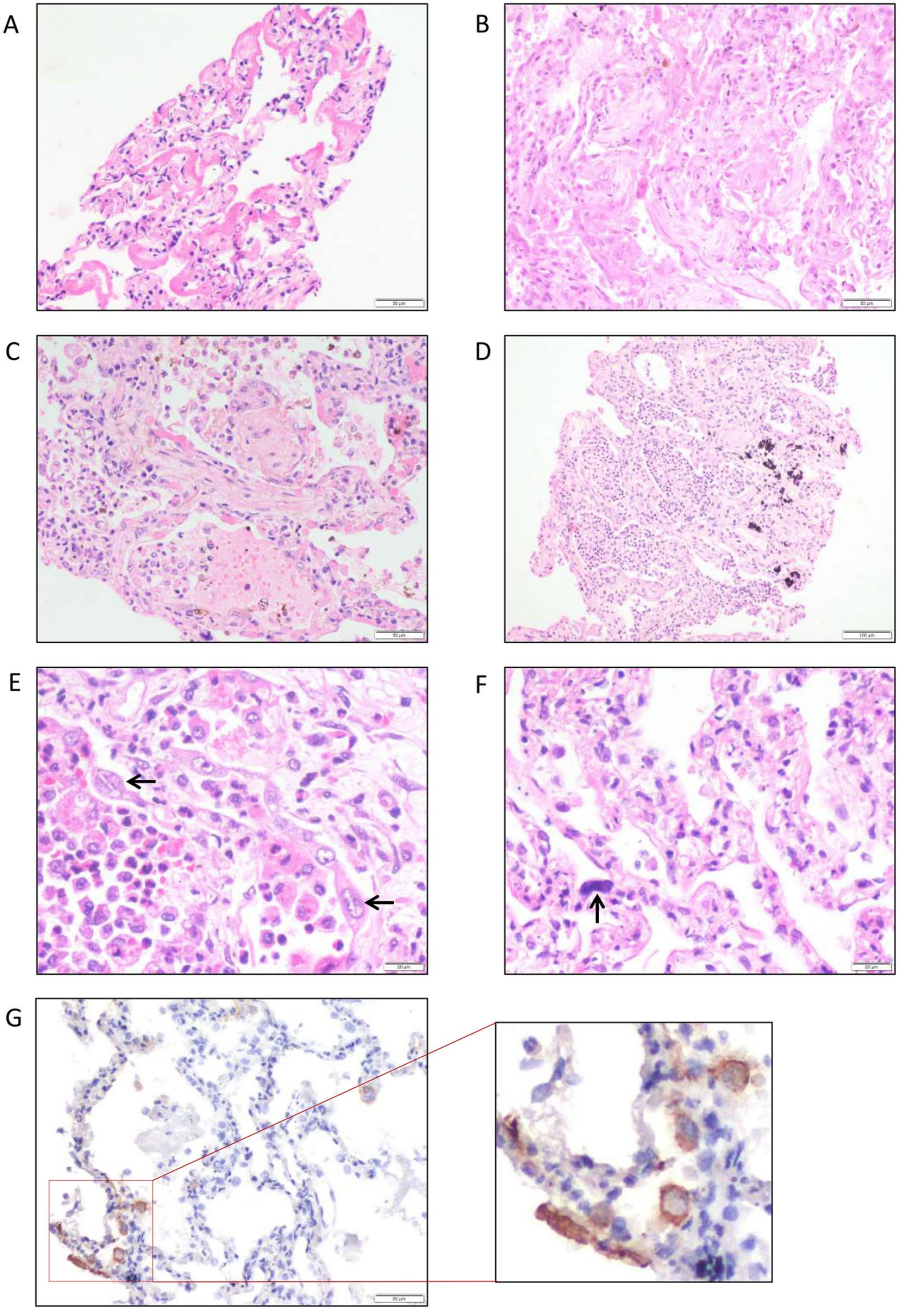
Haematoxylin and eosin-stained sections from representative areas of lung parenchyma infected with COVID-19. The microphotograph shows (**A**) diffuse alveolar damage with the hyaline membrane (exudative phase) (× 200). (**B**) Diffuse alveolar damage with organization (organizing phase) (× 200). (**C**) Organizing pneumonia with interalveolar capillaries filled with microthrombi (× 200). (**D**) Alveolar spaces are filled with neutrophilic infiltrate in a case of organizing DAD (not shown here) indicate superimposed acute bronchopneumonia (× 100). (**E**, **F**) Hyperplastic pneumocytes (**E**, arrow) and megakaryocytes (**F**, arrow) are seen in a case of DAD with acute bronchopneumonia (× 400). (**G**) Representative image of SARS-CoV2-stained tissue from COVID-19 patient. SARS-COV2 IHC shows cytoplasmic granular positivity in pneumocytes.

On histopathology, the majority of cases demonstrated histological evidence of exudative and organising phases of diffuse alveolar damage (69%), acute bronchopneumonia (9%), and acute lung injury (16.3%) (Fig. 1, Table 1, and supplementary Fig. S1). Additionally, two individuals had bacterial microabscesses that were assumed to have developed during hospitalisation. Minority of the cases (5.4%) had no significant histomorphological changes (Table 1).

### SARS-CoV-2 expression

A subset of lung sections (*n* = 10) from COVID-19 patients showed the presence of SARS-CoV2 viral antigen using IHC (Fig. 1B). Among the ten subjects, eight have diffuse alveolar damage on histopathology (Supplementary Table S1).

### Control patients’ clinical data and histopathological findings

**Figure 2:**
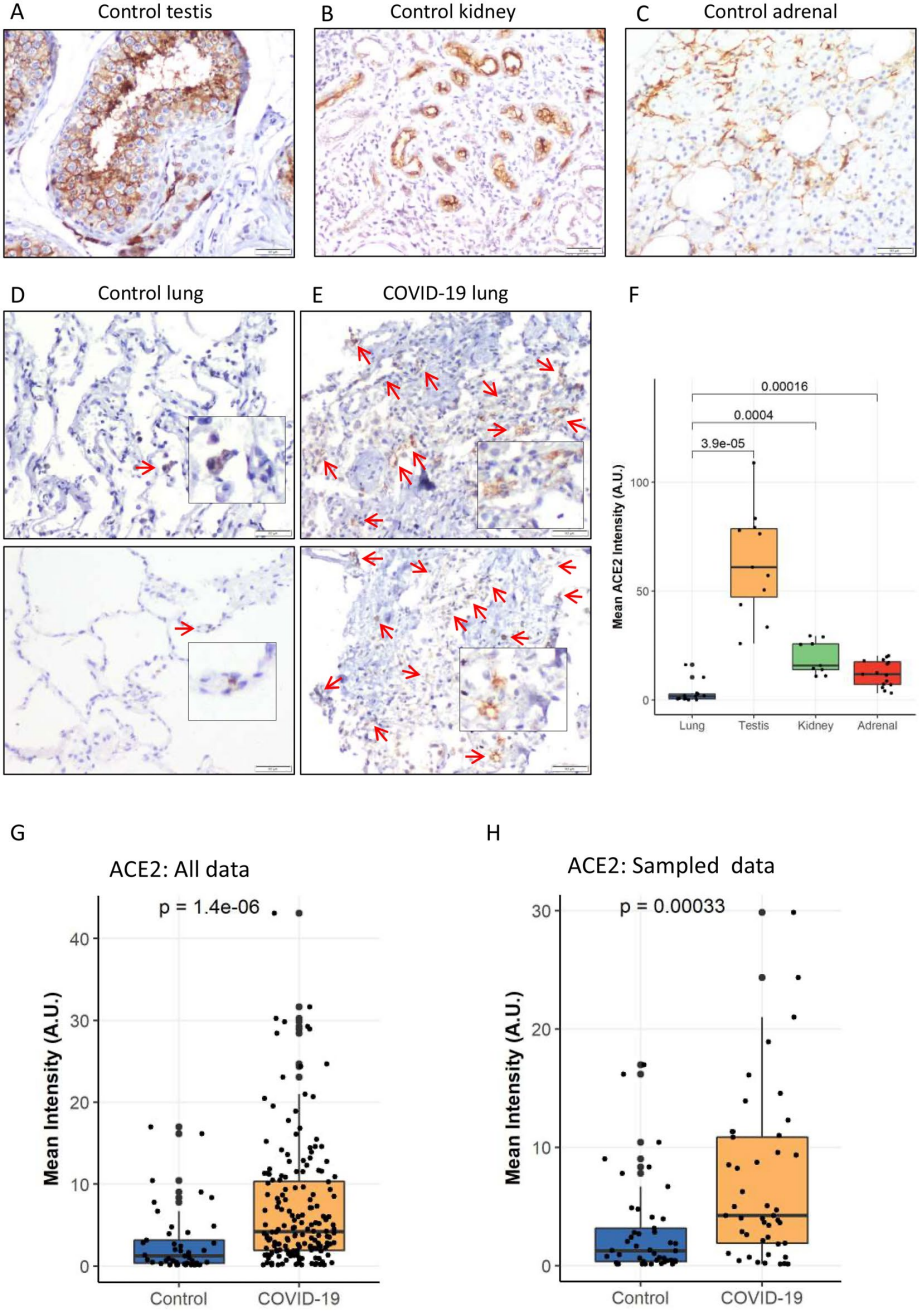
Landscape of ACE2. Large patches of ACE2 positive staining in control tissue of (**A**) testis, (**B**) kidney, and (**C**) in adrenal. Representative images of (**D**) control and (**E**) COVID-19 infected lung sections showing increased ACE2 staining in COVID-19 cases (brown colour, arrows, and insets, **D** and **E**). (**F**) Quantitative analysis of ACE2 IHC intensity in various control tissues. The difference in the mean intensities was calculated using Wilcox test. Significance denoted by exact *P* values (*n* = 13 random images from 15 control lung tissue section, *n* = 11 images from 2 control testis tissue section, *n* = 9 images from 2 control kidney tissue section, *n* = 15 images from 2 control adrenal tissue section). (**G**, **H**) Quantitative analysis of ACE2 IHC intensity in control and COVID-19 lung section (**G**) without sampling analysis (*n* = 45 images from 15 control lung tissue section and *n* = 175 images from 44 COVID-19 lung tissue section) and (**H**) with random sampling analysis (n = 45 images in each group). The difference in the mean intensities was calculated using the Wilcox test, and the exact *P* value denoted significance.

A total of 15 lung sections from control patients were included for comparison. The median age of this cohort was 48 years, with a male-to-female ratio of 1:1 (Table 1). All control patients were either tested negative for SARS-CoV-2 using nasopharyngeal swab PCR or from pre-covid period that is before 2019. The control cohort’s characteristics are summarised in Table 1 and Supplementary Table S2. Histopathology was normal in all control patients, except for two patients whose lung biopsy showed evidence of diffuse alveolar haemorrhage and diffuse alveolar damage (Table 1, Supplementary Table S2). Two sections each of normal kidneys, testes, and adrenal glands were also stained for ACE2 (Fig. 2A–C).

### ACE2 protein expression in cases and controls

ACE2 immunohistochemistry was performed in 44 cases (out of 55) and 15 controls (Supplementary Fig. S1). ACE2 protein was largely undetectable (*n* = 6) or was detected exclusively in the cytoplasm of rare type-II pneumocytes (*n* = 7) in the histologically normal control lungs (Supplementary Table S2). Expression was observed at multiple foci in the two control biopsies that showed diffuse alveolar haemorrhage (DAH) and diffuse alveolar damage (DAD) (Supplementary Table S2). The mean intensity for ACE2 protein was 2.8 ± 0.63 while it was 14.5 and 4.9 in the two cases with DAD and DAH, respectively.

ACE2 protein expression was higher in sections of the normal kidney, testes, and adrenal glands (Fig. 2A–C). Additionally, quantitative imaging analysis revealed that the mean intensity of ACE2 protein is substantially lower in control lung (2.8 ± 0.63) sections than in control kidney (19.5 ± 2.57), testis (66.3 ± 7.38), and adrenal (12.1 ± 1.52) tissues (Fig. 2F).

In lung tissues from COVID-19 patients, the mean ACE2 expression was 7 ± 0.56, with 86% of cases showing a higher mean intensity than the control lung samples, (2.8 ± 0.63) (Fig. 2D,E,G), including one sample with relatively normal histomorphology (3.12 mean intensity). This difference reached strong statistical significance (*P* =  < 0.0001) (Fig. 2G). The higher expression of ACE2 in the COVID-19 lung section was still observed when compared with control lungs from cancer subjects. Random sampling analysis further confirmed the significant increase in ACE2 protein in the lungs of COVID-19 patients (Fig. 2H), negating any bias in the number of samples and images in the control group.

### CD163 and CD61 expression levels

**Figure 3:**
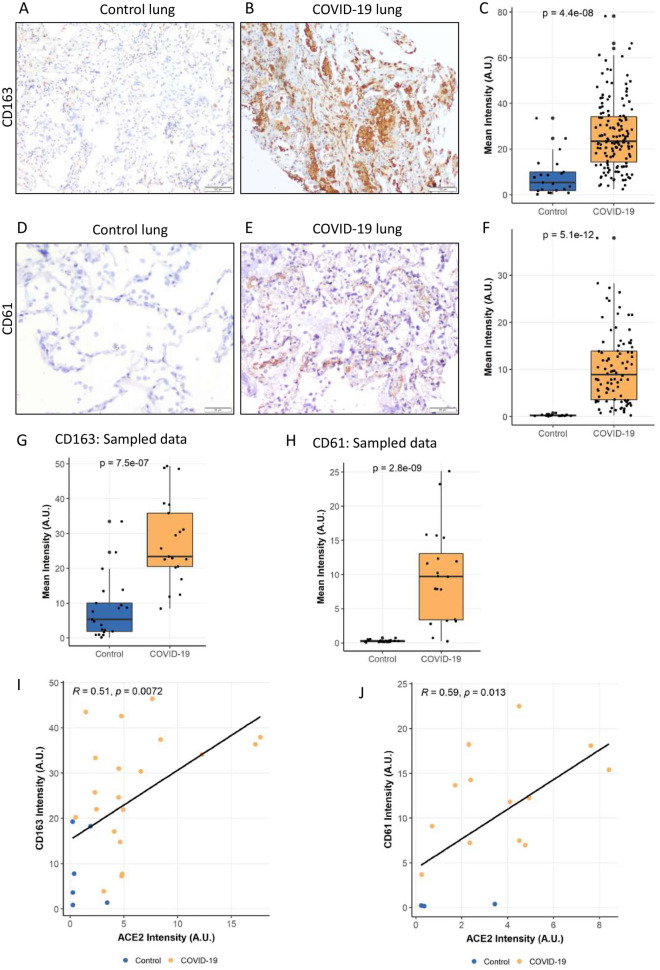
The landscape of CD163 and CD61. Representative images of (**A**) control and (**B**) COVID-19 infected lung sections showing increased CD163 staining in COVID-19 cases, with (**C**) mean Intensities of CD163 in control and COVID-19 samples. The difference in the mean intensities was calculated using Wilcox test Significance denoted by exact *P* values (*n* = 21 images from 6 control lung tissue sections and *n* = 157 images from 36 COVID-19 lung tissue sections). Detection of CD61 protein in representative sections of (**D**) control and (**E**) COVID-19 lung. (**F**) Mean intensity levels of CD61 staining in control and COVID-19 lungs. (*n* = 20 images from 6 control lung tissue section and *n* = 94 images from 26 COVID-19 lung tissue section). (**G**, **H**) Intensities of CD163 (*n* = 21 images/group) and CD61 (*n* = 20 images/group) protein in control and COVID-19 lung section after random sampling analysis. The difference in the mean intensities was calculated using the Wilcox test. Significance denoted by exact *P* values. (**I**, **J**) Correlation between ACE2-CD163 and ACE2-CD61 has been shown. A regression line was calculated using by fitting a linear model to the data. Label at the top shows correlation value (R) and *P* value for the linear fit.

CD163 and CD61 staining were used to examine the inflammatory and coagulopathy components of COVID-19. CD163 and CD61 immunohistochemistry were performed in 36 and 26 cases (Fig. S1) and 6 control samples each of normal lungs, respectively. Among control tissue and cases, macrophage-specific inflammation and platelet plugs and/or platelet-rich microthrombi were found exclusively in COVID-19 lung sections (Fig. 3A–F). Quantitative IHC analysis demonstrated that patients with COVID-19 had significantly higher mean intensities of CD163 (8.5 ± 2.20 vs. 26.3 ± 1.15, *P* =  < 0.0001) and CD61 (0.28 ± 0.04 vs. 10 ± 0.78, *P* =  < 0.0001) staining than those without (Fig. 3C,F). Even after random sampling analysis, CD163 and CD61 protein expression remained significantly higher in COVID-19 patients (Fig. 3G,H). In addition, linear regression analysis revealed a significant association between ACE2 expression and coagulation and inflammatory marker levels in paired samples from cases and controls (*n* = 26 for CD163 and *n* = 16 for CD61) (Fig. 3I,J).

## Discussion

It is well established that the ACE2 protein acts as the entry receptor for the SARS-CoV-2 and the closely related SARS-CoV virus. ACE2 protein expression varies in different organs, and its expression level appears to correlate with the vulnerability of individual organs to infection. In the respiratory tract, ACE2 gene expression levels have been found to be highest in the olfactory bulbs, lower in the nasal respiratory epithelium and least in the bronchioles and alveoli^6,7,14^. At the protein level, however, ACE2 expression is weakly detectable in the lungs and shows poor correlation with mRNA levels^11–13^. By immunohistochemistry, the present study demonstrated negligible expression of the ACE2 protein in normal lung parenchyma. The expression was seen primarily in scattered Type 2 pneumocytes, and its expression is lower when compared to bulk analyses of entire tissues from other organs^6,7,14^. We found higher ACE2 protein levels in kidneys (proximal tubular cells), testis, and adrenal glands (Fig. 2A–C,F), Although not analysed in the present study, cardiac myocytes, endothelial cells, bile duct epithelial cells, and the intestinal mucosal cells are other normal tissues that are known to express ACE2 at high levels and are vulnerable to SARS-CoV-2 infection. Notably, COVID-19 disease presenting with intestinal symptoms or cardiac events is not uncommon. Age and gender-based variations in the amounts of membrane-bound and circulating ACE2 have been reported, with levels being higher in men as compared to women and least in children^15–17^. However, in our small control cohort of mainly adults, we did not find any significant difference in ACE2 protein expression based on gender.

Despite low levels of baseline ACE2 protein expression, the lung parenchyma represents one of the major organs involved in severe COVID-19 disease^8,12,18–21^. The classical RAS activation ACE-Ang II-AT1R axis is known to exacerbate lung damage and inflammation, and a shift toward the ACE2/angiotensin-(1–7)/MAS RAS axis is hypothesised to act as a protective counteractive mechanism^5,6,22,23^. It is also postulated that SARS-CoV-2 infection may decrease the availability of ACE2 for AT1 and ACE degradation, resulting in AngII-dependent lung damage and edema and increased ACE2 levels^5,6,22,23^. In our cohort of patients who had fatal COVID-19 disease, we observed significantly increased ACE2 protein expression levels in the lung parenchyma as compared to control lung tissues (Fig. 2). Notably, all of these tissues showed histological evidence of severe lung parenchymal injury manifested as diffuse alveolar damage (exudative and early proliferative phases) with or without bronchopneumonia and/or microthrombi with no meaningful link between histological severity and ACE intensity, particularly in low and moderate ACE staining cases (Supplementary Fig. S1). A SARS-CoV-2 viral protein was demonstrated in a subset of cases and a linear positive correlation was observed between ACE2 and CD61/CD163 expression levels, representing the inflammatory cascade and vasculopathy in COVID-19 disease, respectively (Fig. 3I,J). These findings regarding the immunopathology and expression of ACE2 in relation to SARS-CoV-2 suggest an abnormal macrophage infiltration and microthrombi response, primarily in the lung, that is not clearly topologically associated with viral presence. The evidence we present suggests that virus-independent immunopathology may be a primary mechanism underlying the fatal COVID-19 infection, supporting personalised therapeutic interventions as a therapeutic strategy^24^. Our results are in line with previous research demonstrating an abnormal macrophage response and plasma-cell abnormalities that are detrimental to the host in COVID-19^11,25–28^ and, more importantly, establish a potential biologic and mechanistic link for future investigation of therapeutic targets underlying fatal COVID-19.

Taken together, all the above findings suggest that ACE2 expression levels are upregulated during severe COVID-19 pulmonary disease. While our study sheds light on the host response to lethal SARS-CoV2 infection, it is not without limitations. Whether ACE2 upregulation directly contributes to disease severity or is a compensatory protective mechanism or just a bystander phenomenon unrelated to disease severity remains unanswered. A comparison among lung tissue from SARS-CoV-2 infected patients with asymptomatic, mild, and severe COVID-19 disease would be ideal for understanding these mechanisms but is beyond the scope of this study. Additionally, information about the patient's medical history, such as specific drugs consumed by the patient (e.g., immunosuppressive agents, antihypertensive agents), may help us determine the likely effect of these medications on the expression of the markers assessed in this study. Unfortunately, we did not have access to all the patient clinical data and thus the analyses of impact of the medical history await further investigation. Moreover, because our analysis also included control lung tissues from biopsies of individuals with certain diseases, we examined only a subset of control autopsy samples (6 of 15) for comparison. As a result, additional studies are required for future in-depth investigations to increase the power of our downstream analysis by accounting for differences in demographic information and disease status.

Given that we found negligible expression of ACE2 in non-COVID-19 lungs irrespective of gender, and a uniform increase in ACE2 expression in all severe COVID-19 lungs, it seems unlikely that baseline pulmonary ACE2 expression levels contribute to the risk of developing COVID-19 pulmonary disease in infected patients. However, expression, correlation, and network analyses have revealed increased levels of ACE2 in the lungs of individuals with respiratory illnesses (COPD, acute respiratory distress syndrome), cardiovascular disease, hypertension, diabetes, chronic renal disease, and cancer^5,6,17,29–33^ and it is well known that individuals with such co-morbidities are susceptible to severe COVID-19 disease, most likely due to increased ACE2 expression, allowing for greater viral invasiveness. Although our control cohort did not include many patients with such co-morbidities, we did notice increased ACE2 expression in two non-COVID19 patients with diffuse alveolar damage and alveolar haemorrhage, supporting that the higher ACE2 expression in diseased lungs may contribute to the increased disease susceptibility and severity^29,33–35^. However, we did not have enough sample size to obtain sufficient statistical power to eliminate false negatives and further studies with larger sample size needs to be conducted to further validate our observations.

To conclude, the present study performed on a relatively large cohort of fatal COVID-19 demonstrates high pulmonary expression of ACE2 protein in their post-mortem lung tissues and negligible expression in control lung tissues, highlighting an important role for ACE2 protein in the pathogenesis of SARS-CoV-2 infection.

## Methods

### Patient sample and tissue processing

The study has been approved by the Institute Ethics Committee of the All India Institute of Medical Sciences New Delhi, India (IEC-536/05.06.2020, OP-05/03.07.2020). All methods in this study were performed in accordance with the guidelines and regulations by the Indian Council of Medical Research (ICMR), the Institute human ethics committee, and hospital for conducting scientific research. The cases comprised patients with pre-mortem PCR-confirmed SARS-CoV-2 infection. After death, minimally invasive post-mortem tissue sampling was performed at a biosafety level 3 post-mortem facility within an average of 2 h after the death. Collection of tissues and data for research was obtained after providing informed written consent from next of kin. Control tissues were obtained from subjects who underwent lung biopsies, surgical resections, or autopsies. In the case of control autopsy, samples were collected from the subject within an average of 2 h after the death in road trauma accidents. For all control subjects, informed consent was obtained from either a personal and/or immediate patient representative for sample collection. Normal kidney, testis, and adrenal tissue were also obtained from the surgical pathology laboratory for comparison of ACE2 expression in different tissue samples. The samples were fixed in formalin and processed into formalin-fixed paraffin-embedded (FFPE) blocks. Blocks were cut into 4-μm sections and stained with haematoxylin and eosin using a standardised procedure.

### Histopathological evaluation

Histopathological evaluations were performed on lung sections stained with haematoxylin and eosin. Two thoracic pathologists (DJ and AN) independently evaluated the slides using Olympus bright-field microscope (Olympus, India). The following features: extent of lung damage, injury, inflammation, presence or absence of hyaline membrane formation, lymphocyte infiltration, organising pneumonia, alveolar fibrin deposition, fibrosis, and histologic features of type 2 pneumocyte hyperplasia, were noted and documented. Images of all assessed tissue sections were captured using CellSens imaging software (Olympus, India) at magnifications of 100 ×, 200 ×, and 400 ×.

### Immunohistochemistry

Immunochemistry was carried out on selected markers, namely, ACE2, CD61, CD163, and SARS-CoV-2 nucleocapsid protein to characterise SARS-CoV-2 entry receptor, platelet component of microthrombi, proinflammatory macrophages, and SARS-CoV-2 levels respectively in the lung parenchyma of patients and control subjects. Depending upon the tissue sample availability, out of a total of 70 lung sections (55 cases and 15 controls), 59 of them (44 cases and 15 controls) were analysed for ACE2, 32 (26 cases and 6 controls) were analysed for CD61, and 42 of them (36 cases and 6 controls) were analysed for CD163. For immunohistochemistry, tissue blocks were cut into 4-μm sections, cleaned in xylene, and hydrated through an alcohol series to distilled water. After antigen retrieval, endogenous peroxidase activity was blocked by treating the sections with 4% hydrogen peroxide for 30 min. Slides were washed with buffer (TRIS Buffer) and blocked with background Protein Block (Mouse and rabbit Specific HRP (ABC) Detection IHC kit ab93677, abcam) for five minutes and washed with buffer. The area around the tissue was marked with a PAP pen. Tissue sections were then incubated overnight with a marker specific primary antibody. The primary antibody concentration was optimised using 3,3'-diaminobenzidine staining and validated to avoid nonspecific staining as follows. ACE-2 antibody (1:400, ACE-2 Recombinant Rabbit Monoclonal Antibody, SN0754, Invitrogen, USA), CD-61 antibody (1:200, CD61 BIO SB Clone-Ep65 RMab, Cell Signaling Technology), CD-163 antibody (1:200, CD163, Monoclonal Antibody (10D6) Invitrogen) and SARS CoV2 antibody (1:100, SARS, Coronavirus 2 Nucleocapsid Monoclonal Antibody (B46F) Invitrogen). After washing, the tissue section was processed with the ABC Detection kit (Mouse and rabbit Specific HRP (ABC) Detection IHC kit ab93677, abcam), according to the manufacturer’s protocol, and HRP activity was detected using the DAB (SignalStrain (R) DAB substrate kit, Cell Signaling Technology). Finally, the sections were counterstained with hematoxylin.

The presence of ACE2, CD163, and CD61 staining of any intensity in the cytoplasm +/− membranous area was defined as positive staining. For the SARS CoV2 detection, its nucleocapsid protein staining of any intensity in the cytoplasm was taken as a positive expression as described previously^36^. Staining was noted in reactive pneumocytes which were lining alveolar walls.

### Immunohistochemistry, imaging and quantification analysis

The staining was examined using an Olympus bright-field microscope at magnifications of 100 × and 200 ×. CellSens imaging software (Olympus, India) was used to photograph the slides. Stains were assessed by imaging the most highly stained portions at least twice across distinct locations, ensuring not to sample the same location more than once. To determine the protein levels, the images captured above at 100 × magnification for CD163 and 200 × magnification for ACE2 and CD61, were then quantified using ImageJ software (National Institutes of Health). A H-DAB colour-based deconvolution tool was used for the separation of DAB staining images. Adjustments were made to the thresholds in order to produce sharp particles and eliminate background noise. The generated picture was then processed to determine the total positive area expressed in terms of mean intensity. The intensity value of each image from a tissue section was considered separately for analysis rather than averaging intensities across all images. This was done to avoid any biologically meaningful normalisation of intensities across images of a tissue section. For grading low, medium, and high intensity, the mean IHC expression values per sample were divided into quartiles. For each group, values up to the 25th quartile were classified as 'low,' those between the 25th and 75th quartiles as 'medium,' and those between the 75th and 100th quartiles as ‘high’.

### Statistical analysis

The power of the study was calculated by keeping one-sided type I error at 0.05. We achieved power of 0.92 (type II error = 0.8) and 0.84 (type II error = 0.16) for CD61 and CD163 intensities, respectively. For ACE2 intensity, the statistical power obtained was 0.5 (type II error = 0.5). The difference between the means of the sample groups was calculated using the Wilcoxon test wherever the data was not normally distributed. A Pearson correlation coefficient was used for estimating correlations and a regression line was drawn using linear fit. Random sampling was performed to account for the imbalance in the number of samples in each sample group. For example, ACE2 samples had 45 intensities values in Control and 180 in COVID-19. To minimise the bias arising due to differences in sample numbers, the same number of intensity values were randomly sampled from the COVID-19 group. The same methodology was employed for the CD163 and CD61 samples. While calculating the correlations, only the samples that had paired data for the groups being compared were selected. Statistical analysis was performed with R 4.0.3 software. *P* < 0.05 was considered to be statistically significant.

## Supplementary Information

Supplementary Information.

## Data Availability

The datasets used and/or analysed during the current study are available from the corresponding author on reasonable request.
