# Supplementary material for: ACE2 protein expression in lung tissues of severe COVID-19 infection
Source: Sci Rep. 2022 Mar 8;12:4058. doi: 10.1038/s41598-022-07918-6 (PMC8902283; doi:10.1038/s41598-022-07918-6)
Supplement: Supplementary file 1 — Supplementary Information. [file 41598_2022_7918_MOESM1_ESM.docx]

**Supplementary information**

**ACE2 protein expression in lung tissues of severe COVID-19 infection**

Atish Gheware^1^, Animesh Ray^2^, Deeksha Rana^1^, Prashant Bajpai^3^, Aruna Nambirajan^1^, S Arulselvi^4^, Purva Mathur^4^, Anjan Trikha^5^, Sudheer Arava^1^, Prasenjit Das^1^, Asit Ranjan Mridha^1^, Geetika Singh^1^, Manish Soneja^2^, Neeraj Nischal^2^, Sanjeev Lalwani^6^, Naveet Wig^2^, Chitra Sarkar^1^, Deepali Jain^1*^

1. Department of Pathology, All India Institute of Medical Sciences, New Delhi, India 110029
2. Department of Medicine, All India Institute of Medical Sciences, New Delhi, India-110029
3. Emory Vaccine Center, International Center for Genetic Engineering and Biotechnology, New Delhi, India- 110067
4. Department of Laboratory Medicine, JPNATC, All India Institute of Medical Sciences, New Delhi, India-110029
5. Department of Anaesthesiology, Critical Care and Pain Medicine, All India Institute of Medical Sciences, New Delhi, India-110029
6. Division of Forensic Pathology and Molecular Laboratory, All India Institute of Medical Sciences, New Delhi, India-110029

***Address for correspondence**

Dr. Deepali Jain, MD, DNB, FIAC

Department of Pathology,

All India Institute of Medical Sciences (AIIMS), New Delhi, India 110029.

Email: [deepalijain76@gmail.com](mailto:deepalijain76@gmail.com)

**Supplementary Tables**

**Table S1.** SARS-CoV-2 IHC positive cases details

| **Case ID** | **Histology** |
| --- | --- |
| 6 | Diffuse alveolar damage |
| 13 | Focal fibrin deposition |
| 15 | Diffuse alveolar damage |
| 17 | Diffuse alveolar damage |
| 22 | Edema fluid accumulation and interalveolar capillaries dilatation |
| 32 | Diffuse alveolar damage |
| 34 | Diffuse alveolar damage |
| 35 | Diffuse alveolar damage |
| 38 | Diffuse alveolar damage |
| 39 | Diffuse alveolar damage |

**Table S2.** Control lung sample details

| **Sample type** | **Diagnosis** | **Average ACE2 intensity (SD)** |
| --- | --- | --- |
| Autopsy (*n*=6) | Road trauma accident | 1.06 (1.57) |
| Biopsy (*n*=2) | Intra-alveolar haemorrhage (*n*=1) and diffuse alveolar damage (*n*=1). | 9.75 (6.74) |
| Surgical resection (*n*=7) | Adjacent normal lung parenchyma in cases of squamous cell carcinoma (*n*=2), invasive mucinous adenocarcinoma (*n*=1), adenocarcinoma (*n*=2), typical carcinoid (*n*=2) | 2.51 (2.84) |

**Supplementary figure**

**
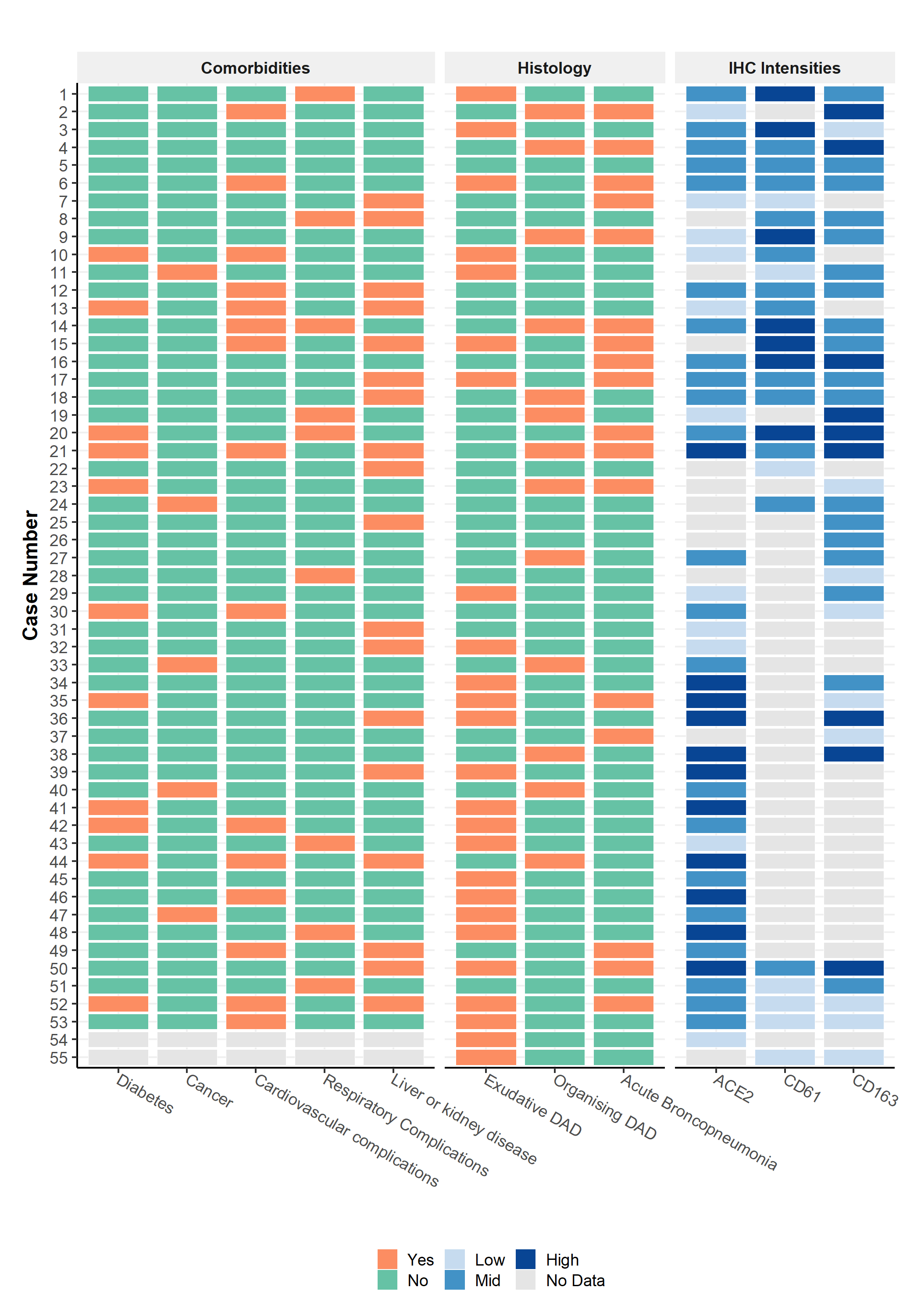
**

**Figure S1. Summary of COVID-19 patients.** Heatmap representing coexisting comorbidity, histopathological changes and IHC experimental details of COVID-19 patients.
